# Synthesis, Enantiomeric Resolution and Biological Evaluation of HIV Capsid Inhibition Activity for Racemic, (*S*)- and (*R*)-PF74

**DOI:** 10.3390/molecules26133919

**Published:** 2021-06-26

**Authors:** Stuart Ruddell, Elena Sugrue, Sarah Memarzadeh, Lorna Mae Hellam, Sam J. Wilson, David J. France

**Affiliations:** 1 School of Chemistry, University of Glasgow, Glasgow G12 8QQ, UK; stuart.ruddell@tum.de (S.R.); s.memarzadeh.1@research.gla.ac.uk (S.M.); l.hellam.1@research.gla.ac.uk (L.M.H.); 2 MRC-University of Glasgow Centre for Virus Research, Institute of Infection, Immunity and Inflammation, University of Glasgow, Glasgow G12 8TA, UK; elena.sugrue@glasgow.ac.uk

**Keywords:** antiviral, stereochemistry, racemization, docking

## Abstract

PF74 is a capsid-targeting inhibitor of HIV replication that effectively perturbs the highly sensitive viral uncoating process. A lack of information regarding the optical purity (enantiomeric excess) of the single stereogenic centre of PF74 has resulted in ambiguity as to the potency of different samples of this compound. Herein is described the synthesis of enantiomerically enriched (*S*)- and (*R*)-PF74 and further enrichment of the samples (≥98%) using chiral HPLC resolution. The biological activities of each enantiomer were then evaluated, which determined (*S*)-PF74 (IC_50_ 1.5 µM) to be significantly more active than (*R*)-PF74 (IC_50_ 19 µM). Computational docking studies were then conducted to rationalise this large discrepancy in activity, which indicated different binding conformations for each enantiomer. The binding energy of the conformation adopted by the more active (*S*)-PF74 (ΔG = −73.8 kcal/mol) was calculated to be more favourable than the conformation adopted by the less active (*R*)-enantiomer (ΔG = −55.8 kcal/mol) in agreement with experimental observations.

## 1. Introduction

Human Immunodeficiency Virus (HIV) is a pathogenic retrovirus that infects cells of the human immune system and, if left untreated, can result in Acquired Immunodeficiency Syndrome (AIDS). An estimated 38 million people are infected worldwide and, since the epidemic began in the early 1980s, approximately 33 million people have died from HIV-related deaths [1,2]. Combination antiretroviral therapy (cART) currently provides effective viral suppression for most patients, but concerns including drug resistance and adverse drug events provide a powerful motivation for investigation into new mechanisms to treat HIV-1 infection [3].

**Figure 1:**
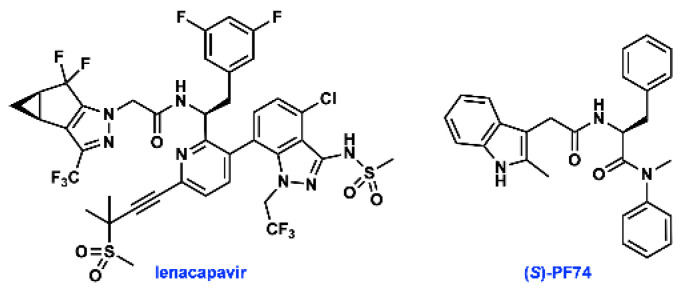
Structures of lenacapavir and (*S*)-PF74.

The HIV-1 capsid protein (CA) multimerizes to form a fullerene-like shell that encapsidates the viral RNA genome and is essential for both early and late stages of the viral replication cycle [4]. Small molecule ligands that target CA have been identified, highlighted by lenacapavir (GS-6207, Figure 1), which is currently in phase 2/3 clinical trials. The small molecule PF74 was the first to be co-crystallised with CA, and has been widely studied [5,6,7]. PF74 has been shown to both stabilise and destabilise the HIV capsid, but the precise mechanism by which PF74 exerts its antiviral effect has not been fully established. Regardless of whether the stabilising or destabilising effect is more important, interfering with capsid stability perturbs the highly sensitive uncoating process, and thus inhibits HIV infection [6,8,9,10,11].

PF74 is a non-natural derivative of phenylalanine, a sub-structure that has been further optimised in lenacapavir. However, crucial information regarding the stereogenic centre present in PF74 is scarce in published syntheses and studies on the biological activities of this compound, and is not readily available from commercial suppliers of PF74 [5,6,12,13,14]. As a derivative of phenylalanine, PF74 is prepared from enantioenriched material from natural sources. Although there is a vast body of literature on strategies to avoid epimerisation during peptide coupling [15], the inherent asymmetry of biological systems necessitates that control of absolute stereochemical configuration of small molecule inhibitors remains integral to their activity [14,16,17,18].

The scope of this project was to study stereochemical integrity in the synthesis of PF74, and evaluate the biological activity of the two enantiomers in both cell assay and through computational docking.

## 2. Results and Discussion

### 2.1. Synthesis and Characterisation

**Scheme 1:**
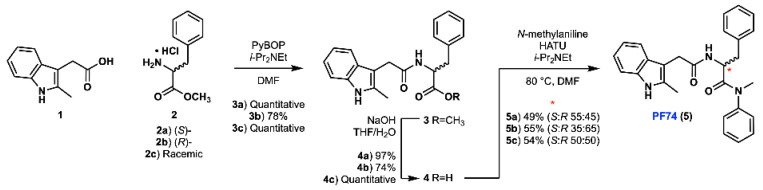
Synthesis of PF74 in three different enantiomer ratios.

Our improved synthesis of PF74 was conducted based on a previously reported literature synthesis, with some optimisation [13]. Due to the likelihood of epimerisation in this route, we chose to synthesise (*S*)-, (*R*)- and racemic PF74 using enantiomerically enriched starting materials, which allowed us to assess any epimerisation and to compare our synthetic material with commercially available PF74. A PyBop-mediated amide coupling between 2-methylindole-3-acetic acid (**1**) and either (*S*)-, (*R*)- or racemic phenylalanine methyl ester hydrochloride (**2a**, **2b** and **2c,** respectively) was utilised to generate the amide containing methyl ester **3** (Scheme 1). Yields for these amidations were greater when using PyBop than those obtained using HATU [13]. Following this amidation, saponification of methyl ester **3** to acid **4** was conducted. Subsequent HATU-mediated amide coupling of acid **4** (**a**, **b** and **c**) with *N*-methylaniline generated PF74 (**5a**, **5b** and **5c**).

**Figure 2:**
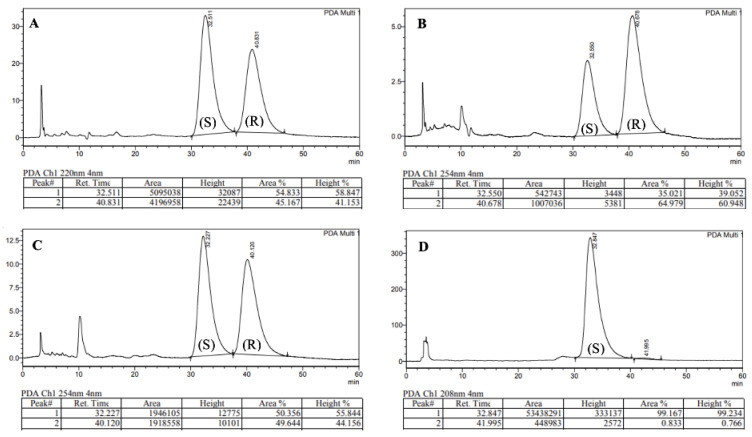
Chiral HPLC traces of: (**A**) **5a**, (**B**) **5b**, (**C**) **5c**, (**D**) Enantiomerically enriched (*S*)-PF74.

While extensive literature precedent suggests that stereochemical integrity should be maintained during the coupling and hydrolysis protocols to form **4** [15], the poor nucleophilicity of *N*-methylaniline posed a significant risk of epimerisation during the formation of tertiary amide **5** [19]. As a result of the sterically hindered and electronically deactivated nature of this secondary aniline, the lifetime of the activated ester intermediate (formed upon reaction of acid **4** with HATU) is extended and elevated temperatures were required (80 °C). The combination of these two factors greatly increases the likelihood of oxazolone-mediated epimerisation [20,21]. In order to investigate the occurrence of epimerisation under our reaction conditions, analytical chiral HPLC was utilised (Figure 2). This analysis confirmed that significant epimerisation was present in each of the syntheses of PF74 (**5a** and **5b**). Consequently, given the variable erosion of stereochemical integrity we observed and the similarity between our route and the chosen precedent, this implied that the previously reported synthesis also produced PF74 with ambiguous optical purity.

To assess the integrity of the stereogenic carbon in commercial PF74 (as this information is typically unavailable), two samples were purchased and analysed using chiral HPLC. This revealed a high degree of variability, with one sample being highly enantioenriched (*er* 99:1, Figure 2D), the other nearly racemic (*S*:*R* 51:49). With this observation, it seemed plausible that the hitherto reported biological activities of PF74 used either racemic PF74 or PF74 with ambiguous enantiomeric ratios. With the aim of testing the biological activity of each enantiomer of PF74, enantiomeric resolution was conducted using chiral HPLC. Using this approach, 1 mg each of highly enantioenriched (*S*)-PF74 (*S*:*R* 99.2:0.8) and (*R*)-PF74 (*S*:*R* 1.6:98.4) was generated.

### 2.2. Biological Evaluation

#### 2.2.1. Antiviral Activity

**Figure 3:**
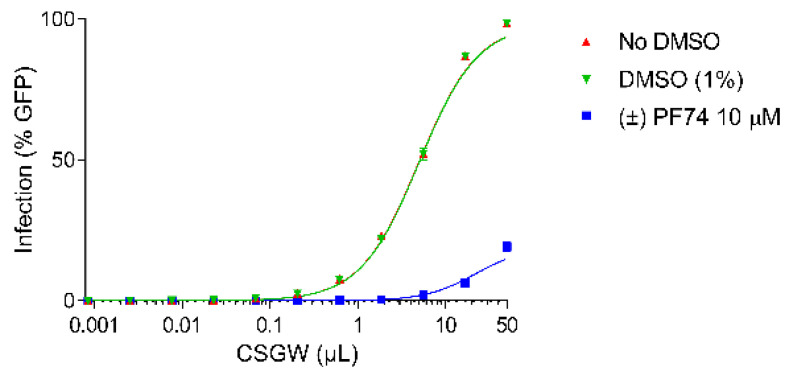
CSGW infection of HEK 293T cells in the presence of no compound (red), DMSO vehicle (green) and 10 µM racemic PF74 (blue).

Activity assays were conducted in the Human Embryonic Kidney cell line (HEK293T) using a VSV G protein (VSV-G) pseudotyped single cycle HIV-1 reporter system (CSGW) [22]. Use of this reporter system enables quantification of infected cells using flow cytometry, where the percentage of GFP positive cells is used as the metric of infection. By titrating CSGW on HEK293T cells, the baseline infectivity of the virus (in the absence of PF74) was established (e.g., 1 µL CSGW infected ca. 10% of cells) (Figure 3, red). The addition of DMSO (vehicle, negative control) to the assay had no effect on the infectivity of the virus (Figure 3, green). Additionally, when the cells were treated with 10 µM synthetic (racemic) PF74, effective inhibition of viral infectivity was observed, as indicated by decrease in the percentage of cells infected for a given dose of CSGW (Figure 3, blue).

**Figure 4:**
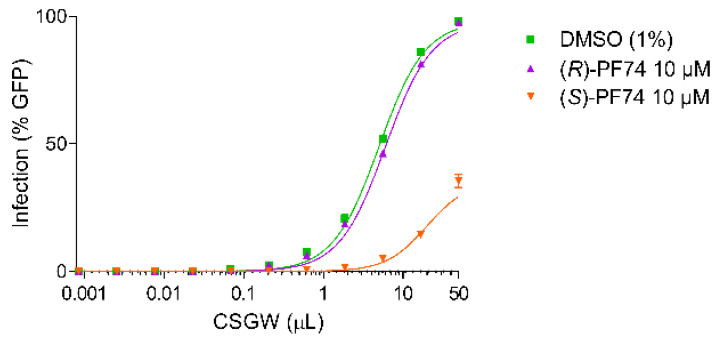
CSGW infection of HEK293T cells in the presence of DMSO vehicle (green), 10 µM (*R*)-PF74 (purple) and 10 µM (*S*)-PF74 (orange).

When the cells were treated with the highly enantiomerically enriched enantiomers of PF74, differential activities were observed (Figure 4). With (*R*)-PF74 no significant inhibition of viral infectivity was observed above that of the baseline CSGW infectivity. With (*S*)-PF74 however, significant inhibition of viral infectivity was observed, indicating (*S*)-PF74 to be considerably more active.

**Figure 5:**
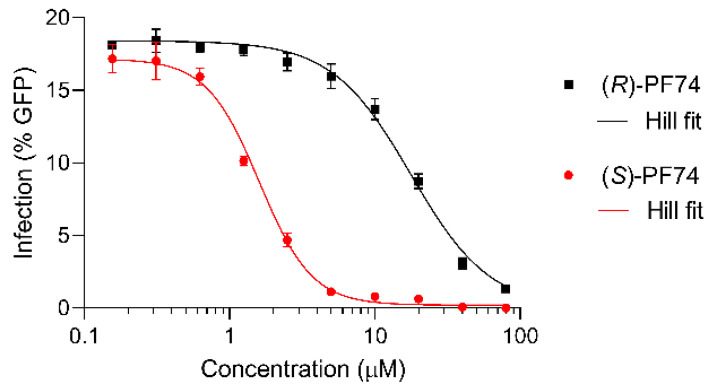
Dose response assays of (*R*)- and (*S*)-PF74 (black and red, respectively) for IC_50_ determination, plotted with Hill equation fit lines (grey).

To quantify this difference more conclusively, IC_50_ values were determined for each of the enantiomers (Figure 5). For this dose response assay, a fixed amount of virus was added (to produce approximately 15–20% cell infection) and a titration of each enantiomer of PF74 was administered to the cells. The results were fitted to the Hill equation and plotted. (*S*)-PF74 was determined to have an IC_50_ of 1.5 µM and the less active (*R*)-PF74 an IC_50_ of 19 µM (Figure 5).

#### 2.2.2. Docking Studies

**Figure 6:**
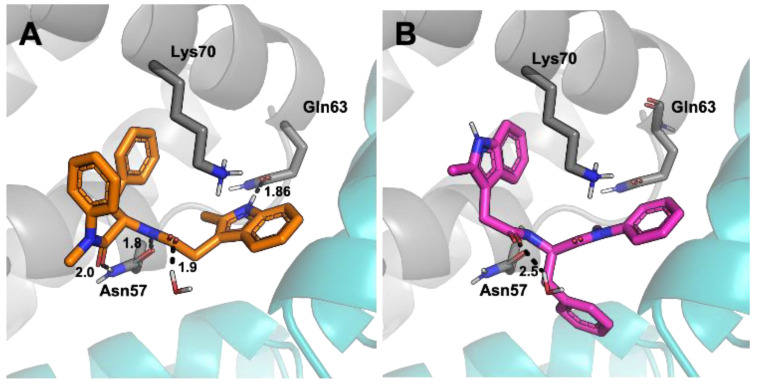
View of CA residues with potential interactions with PF74 in the binding pocket, with hydrogen bonds shown as dotted black lines. Numbers indicate bond lengths in ångströms. (**A**) (*S*)-PF74, (**B**) (*R*)-PF74.

To rationalise the difference in efficacy between the two enantiomers, docking studies were conducted using the Schrödinger suite. (*S*)-PF74 binding at the CA hexamer binding site was shown to have seven potential favourable interactions. These include π-cation interactions between the indole and benzyl groups both to Lys70 as well as hydrogen bonding interactions from the indole to Gln63, and between Asn57 and the phenylalanine sub-structure embedded in PF74 (Figure 6A). Three locations of unfavourable solvent exposure were also identified, all in the region of the *N*-methyl aniline of PF74. These findings closely matched the literature reported binding interactions of the PF74-capsid crystal structure, thus generating high confidence in the chosen method [6]. Additionally, the binding energy of (*S*)-PF74 was calculated using a MMGBSA function and determined to have a ΔG of −73.8 kcal/mol. Docking of (*R*)-PF74 showed an alternative conformation to that observed for (*S*)-PF74. This conformation indicated only four potential favourable interactions comprised of three π-cation interactions to Lys70 and a single water-mediated hydrogen bonding interaction (Figure 6B). Three sites of unfavourable solvent exposure for the *R*-enantiomer were also identified, two on the phenylalanine phenyl component of PF74, and one on the indole methyl group. The binding energy of (*R*)-PF74 was calculated using the same MMGBSA function and determined to have a ΔG of −55.8 kcal/mol.

The more favourable calculated binding conformation of (*S*)-PF74 corroborates the experimental observation that (*S*)-PF74 was a more potent HIV inhibitor than (*R*)-PF74. Therefore, the results of these computational studies constitute a reasonable explanation as to why the efficacy of (*S*)-PF74 was approximately 15-fold greater than that of (*R*)-PF74.

## 3. Materials and Methods

### 3.1. General Chemical Methods 

All reagents were purchased from commercial suppliers and used without further purification unless otherwise stated. Reactions involving air-sensitive agents and dry solvents were performed in glassware that had been dried in an oven (150 °C) or flame-dried in vacuo and allowed to cool in vacuo before being flushed with argon. These reactions were carried out with the exclusion of air using an argon atmosphere. Reactions were monitored by thin layer chromatography (TLC, Merck, Darmstadt, Germany) on silica gel 60 covered aluminium sheets. TLC plates were developed under UV-light and/or with an acidic ethanolic anisaldehyde solution or a KMnO_4_ solution. NMR spectra (see Supplementary Materials) were recorded on a DPX-400 spectrometer (^1^H NMR at 400 MHz, ^13^C NMR at 100 MHz) (Bruker, Billerica, MA, USA) or a DPX-500 spectrometer (^1^H NMR at 500 MHz and ^13^C NMR at 125 MHz) (Bruker, Billerica, MA, USA). Chemical shifts are reported in ppm. ^1^H NMR spectra were recorded with CDCl_3_ or CD_3_OD as the solvent using residual CHCl_3_ (δ = 7.26) or CHD_2_OD (δ = 3.31) as internal standard, and for ^13^C NMR spectra the chemical shifts are reported relative to the central resonance of CDCl_3_ (δ = 77.16) or CD_3_OD (δ = 49.00). Signals in NMR spectra are described as singlet (s), doublet (d), triplet (t), quartet (q), multiplet (m), broad (br) or a combination of these, which refers to the spin–spin coupling pattern observed. Spin–spin coupling constants are reported in Hertz (Hz) and are uncorrected. Two-dimensional NMR spectroscopy (COSY, HSQC, HMBC, NOESY) and ^13^C DEPT NMR spectroscopy were used where appropriate to assist the assignment of signals in the ^1^H and ^13^C NMR spectra. IR spectra were obtained employing a FTIR-8400 instrument (Shimadzu, Kyoto, Japan) with a Golden Gate™ attachment that uses a type IIa diamond as a single reflection element so that the IR spectrum of the compound (solid or liquid) could be detected directly (thin layer). Melting point ranges were collected using a Barnstead Electrothermal 9100 melting point apparatus (Thermo Fisher Scientific, Waltham, MA, USA). High resolution mass spectra were recorded under ESI or EI conditions by the analytical services at the University of Glasgow. Liquid chromatography-mass spectrometry was conducted using a Dionex UltiMate 3000 LC system (Thermo Fisher Scientific, Waltham, MA, USA) coupled with a LCQ Fleet ion trap mass spectrometer (Thermo Fisher Scientific, Waltham, MA, USA). A Dr Maisch GmbH Reprosil Gold 120 C18 3 µm 150 × 4 mm column was used with UV absorption detected at 214 nm. A linear gradient of 5–95% HPLC grade acetonitrile in ultrapure water with 0.1% trifluoroacetic acid over 10 or 40 min was utilised with a flow rate of 1 mL min^−1^. Chiral HPLC analysis was conducted using a LC-20AD prominence liquid chromatograph (Shimadzu, Kyoto, Japan) with a CBM-20A prominence communications bus module, a DGU-20A5 prominence degasser and a SPD-M20A prominence diode array detector. A Shimadzu CTO-20AC prominence column oven was utilised in combination with Diacel Chemical Industries chiralcel OD-H 5 μm 4.6 × 250 mm reverse phase analytical column with an isocratic mobile phase of HPLC grade isopropyl alcohol in HPLC grade hexane.

### 3.2. Compound Synthesis and Characterization

#### 3.2.1. General Procedure to Methyl Ester (**3**)

Phenylalanine methyl ester hydrochloride **2** (1.05 equiv), PyBOP (1 equiv.) and *N*,*N*-diisopropylethylamine (3 equiv.) were added to a solution of 2-methyl-3-indoleacetic acid **1** (1 equiv.) in *N*,*N*-dimethylformamide (0.4 M). The resulting solution was stirred at room temperature for 18 h. The crude mixture was diluted with water and extracted with ethyl acetate. The combined organic layers were washed with 5% aqueous lithium chloride, dried over magnesium sulfate, filtered, and concentrated in vacuo. The product was purified by column chromatography on silica gel using an eluent of 5–20% ethyl acetate in dichloromethane.

*Methyl (2-(2-Methyl-1H-indol-3-yl)acetyl)-l-phenylalaninate* (**3a**)

Following the general procedure described above, 12.2 g (quantitative) of **3a** was obtained as a viscous oil. 

^1^H NMR (400 MHz, CDCl_3_) δ 7.88 (1H, s, Ar-NH), 7.42 (1H, d, *J* = 7.8, Ar-H), 7.31 (1H, d, *J* = 7.9, Ar-H), 7.18 (1H, t, *J* = 7.5, Ar-H), 7.13 (1H, d, *J* = 7.8, Ar-H), 7.09 (1H, d, *J* = 7.9, Ar-H), 7.00 (2H, t, *J* = 7.5, 2 × Ar-H), 6.71 (2H, d, *J* = 7.5, 2 × Ar-H), 5.99 (1H, d, *J* = 7.8, -CONH), 4.84 (1H, dt, *J* = 6.6, 6.6, -CH), 3.64 (2H, s, -COCH_2_),), 3.64 (3H, s, -CH_3_), 2.97 (1H, dd, *J* = 13.9, 5.4, -CH_2_), 2.91 (1H, dd, *J* = 13.8, 5.5, -CH_2_), 2.29 (3H, s, -CH_3_).

^13^C NMR (101 MHz, CDCl_3_) δ 171.9 (-CONH), 171.1 (-COOR), 135.6 (Ar-C), 135.5 (Ar-C), 133.4 (Ar-C), 129.2 (2 × Ar-CH), 128.5 (2 × Ar-CH), 128.4 (Ar-C), 127.0 (Ar-CH), 121.9 (Ar-CH), 120.2 (Ar-CH), 118.0 (Ar-CH), 110.5 (Ar-CH), 104.7 (Ar-C), 52.8 (-CH), 52.3 (-OCH_3_), 37.7 (-CH_2_), 32.2 (-CH_2_), 11.6 (-CH_3_).

HRMS (ESI) exact mass calculated for C_21_H_22_N_2_O_3_Na [M + Na]^+^ *m*/*z* 373.1523, found *m*/*z* 373.1514. IR (thin film) 2952, 1741, 1651.

*Methyl (2-(2-Methyl-1H-indol-3-yl)acetyl)-d-phenylalaninate* (**3b**)

Following the general procedure outlined above, 719 mg (78%) of compound **3b** was obtained as a viscous oil. 

^1^H NMR (400 MHz, CDCl_3_) δ 7.92 (1H, s, Ar-NH), 7.42 (1H, d, *J* = 7.7, Ar-H), 7.31 (1H, d, *J* = 7.7, Ar-H), 7.18 (1H, t, *J* = 6.8, Ar-H), 7.13 (1H, d, *J* = 7.8, Ar-H), 7.09 (1H, t, *J* = 7.8, Ar-H), 6.09 (2H, t, *J* = 6.7, 2 × Ar-H), 6.70 (2H, d, *J* = 6.5, 2 × Ar-H), 6.00 (1H, d, *J* = 8.3, -CONH), 4.90–4.76 (1H, m, -CH), 3.64 (2H, s, -CH_2_), 3.64 (3H, s, -CH_3_), 3.03–2.85 (2H, m, -CH_2_), 2.28 (3H, s, -CH_3_). 

^13^C NMR (101 MHz, CDCl_3_) δ 171.9 (-CONH), 171.1 (-COOR), 135.6 (Ar-C), 135.5 (Ar-C), 133.4 (Ar-C), 129.1 (2 × Ar-CH), 128.5 (2 × Ar-CH), 128.4 (Ar-C), 127.0 (Ar-CH), 121.9 (Ar-CH), 120.2 (Ar-CH), 118.0 (Ar-CH), 110.5 (Ar-CH), 104.6 (Ar-C), 52.8 (-CH), 52.3 (-OCH_3_), 37.7 (-CH_2_), 32.1 (-CH_2_), 11.6 (-CH_3_). 

HRMS (ESI) exact mass calculated for C_21_H_22_N_2_O_3_Na [M + Na]^+^ *m*/*z* 373.1523, found *m*/*z* 373.1514. IR (thin film) 2952, 1741, 1651.

*Methyl (2-(2-Methyl-1H-indol-3-yl)acetyl)-dl-phenylalaninate* (**3c**)

Following the general procedure described above, 925 mg (quantitative) of **3c** was obtained.

^1^H NMR (400 MHz, CDCl_3_) δ 7.89 (1H, s, Ar-NH), 7.42 (1H, d, *J* = 7.8, Ar-H), 7.31 (1H, d, *J* = 7.9, Ar-H), 7.18 (1H, t, *J* = 7.7, Ar-H), 7.15–7.11 (1H, m, Ar-H), 7.11–7.05 (1H, m, Ar-H), 7.00 (2H, t, *J* = 7.7, 2 × Ar-H), 6.70 (2H, d, *J* = 7.5, 2 × Ar-H), 5.99 (1H, d, *J* = 8.3, -CONH), 4.88–4.80 (1H, m, -CH), 3.64 (2H, s, -CH_2_), 3.64 (3H, s, -CH_3_), 3.00–2.86 (2H, m, -CH_2_), 2.28 (3H, s, -CH_3_). 

^13^C NMR (101 MHz, CDCl_3_) δ 171.9 (-CONH), 171.1 (-COOR), 135.6 (Ar-C), 135.5 (Ar-C), 133.4 (Ar-C), 129.2 (2 × Ar-CH), 128.5 (2 × Ar-CH), 128.4 (Ar-C), 127.0 (Ar-CH), 121.9 (Ar-CH), 120.2 (Ar-CH), 118.0 (Ar-CH), 110.5 (Ar-CH), 104.7 (Ar-C), 52.8 (-CH), 52.3 (-OCH_3_), 37.7 (-CH_2_), 32.2 (-CH_2_), 11.6 (-CH_3_). 

HRMS (ESI) exact mass calculated for C_21_H_22_N_2_O_3_Na [M + Na]^+^ *m*/*z* 373.1523, found *m*/*z* 373.1514. IR (thin film) 2952, 1741, 1651. Melting point 126–130 °C.

#### 3.2.2. General Procedure to Acid (**4**)

To a stirred solution of **3** (1 equiv) in tetrahydrofuran (0.23 M) and water (0.34 M) was added a solution of sodium hydroxide (1.5 equiv) in methanol (1 M NaOH) and the resulting solution was stirred vigorously at room temperature for 18 h. The reaction mixture was diluted with water and the crude biphasic mixture was washed with dichloromethane to remove organic impurities. The aqueous phase was then acidified with 2 M aqueous hydrochloric acid forming a thick white precipitate. The aqueous phase was extracted with dichloromethane and the combined organic phases were dried with magnesium sulfate, filtered, and concentrated in vacuo. The compound was used without further purification.

*(2-(2-Methyl-1H-indol-3-yl)acetyl)-d-phenylalanine* (**4a**)

Following the general procedure described above, 989 mg (97%) of **4a** was obtained. 

^1^H NMR (400 MHz, CDCl_3_) δ 8.14 (1H, s, Ar-NH), 7.34 (1H, d, *J* = 7.8, Ar-CH), 7.26 (1H, d, *J* = 7.0, Ar-CH), 7.16 (1H, t, *J* = 7.1, Ar-CH), 7.09 (1H, t, *J* = 7.7, Ar-CH), 7.09 (1H, t, *J* = 7.7, Ar-CH), 6.99 (2H, t, *J* = 7.6, 2 × Ar-CH), 6.74 (2H, d, *J* = 7.1, 2 × Ar-CH), 6.15 (1H, d, *J* = 7.9, -CONH), 4.76 (1H, dd, *J* = 13.1, 6.7, -CH), 3.61 (2H, s, -CH_2_), 3.00 (1H, dd, *J* = 13.9, 5.2, -CH_2_), 2.89 (1H, dd, *J* = 13.9, 6.7, -CH_2_), 2.13 (3H, s, -CH_3_). 

^13^C NMR (101 MHz, CDCl_3_) δ = 174.0 (-COOH), 172.9 (-CONH), 135.5 (Ar-C), 135.4 (Ar-C), 133.8 (Ar-C), 129.2 (2 × Ar-CH), 128.6 (2 × Ar-CH), 128.2 (Ar-C), 127.0 (Ar-CH), 121.8 (Ar-CH), 120.1 (Ar-CH), 117.8 (Ar-CH), 110.7 (Ar-CH), 103.7 (Ar-C), 53.2 (-CH), 37.0 (-CH_2_), 31.8 (-CH_2_), 11.4 (-CH_3_). 

HRMS (ESI) exact mass calculated for C_20_H_20_N_2_O_3_Na [M + Na]^+^ *m*/*z* 359.1366, found *m*/*z* 359.1355. IR (thin film) 2916, 1724, 1618.

*(2-(2-Methyl-1H-indol-3-yl)acetyl)-l-phenylalanine* (**4b**)

Following the general procedure outlined above, 358 mg (74%) of **4b** was obtained as a solid. 

^1^H NMR (400 MHz, CDCl_3_) δ 7.89 (1H, s, Ar-NH), 7.36–7.29 (2H, m, 2 × Ar-CH), 7.18 (1H, t, *J* = 7.4, Ar-CH), 7.13 (1H, t, *J* = 8.3, Ar-CH), 7.11 (1H, t, *J* = 8.3, Ar-CH), 7.07–7.00 (2H, m, 2 × Ar-CH), 6.75 (2H, d, *J* = 7.5,2 × Ar-CH), 5.99 (1H, br s, -CONH), 4.69 (1H, br s, -CH), 3.64 (2H, br s, -CH_2_), 3.04 (1H, br dd, *J* = 13.8, 5.5, -CH_2_), 2.94 (1H, br dd, *J* = 13.8, 5.5, -CH_2_), 2.20 (3H, s, -CH_3_). 

^13^C NMR (101 MHz, CDCl_3_) δ = 174.0 (-COOH), 172.9 (-CONH), 135.5 (Ar-C), 135.4 (Ar-C), 133.8 (Ar-C), 129.2 (2 × Ar-CH), 128.6 (2 × Ar-CH), 128.2 (Ar-C), 127.0 (Ar-CH), 121.8 (Ar-CH), 120.1 (Ar-CH), 117.8 (Ar-CH), 110.7 (Ar-CH), 103.7 (Ar-C), 53.2 (-CH), 37.0 (-CH_2_), 31.8 (-CH_2_), 11.4 (-CH_3_). 

HRMS (ESI) exact mass calculated for C_20_H_20_N_2_O_3_Na [M + Na]^+^ *m*/*z* 359.1366, found *m*/*z* 359.1355. IR (thin film) 2916, 1724, 1618. Melting point 140–143 °C.

*(2-(2-Methyl-1H-indol-3-yl)acetyl)-d-phenylalanine* (**4c**)

Following the general procedure outlined above, 491 mg (quantitative) of **4c** was obtained.

Spectroscopic data matched that of acid **4a**.

#### 3.2.3. Preparation of PF74 (**5**)

PF74 was prepared according to a modified literature procedure [13]. To a stirred solution of acid **4** (100 mg, 0.300 mmol) in *N*,*N*-dimethylformamide (0.3 mL) was added *N*-methylaniline (49 µL, 0.45 mmol), HATU (171 mg, 0.450 mmol) and *N*,*N*-diisopropylethylamine (0.11 mL, 0.60 mmol) and the resulting solution was heated to 65 °C and stirred for 48 h. To the reaction mixture was added dichloromethane and half saturated aqueous sodium hydrogen carbonate solution and the aqueous phase was extracted with dichloromethane. The combined organic phases were washed with 5% aqueous lithium chloride solution, dried over magnesium sulfate, filtered, and concentrated in vacuo. The crude mixture was purified by column chromatography on silica gel using an eluent of 1–5% ethanol in dichloromethane.

*Natural (S)-PF74* (**5a**)

Following the general procedure described above, 63 mg (49%) of **5a** was obtained.

^1^H NMR (400 MHz, CDCl_3_) δ 8.26 (1H, s, Ar-NH), 7.39–7.31 (4H, m, 4 × Ar-CH), 7.26 (1H, d, *J* = 8.0, Ar-CH), 7.18–7.04 (5H, m, 5 × Ar-CH), 6.97–6.89 (2H, m, 2 × Ar-CH), 6.71–6.65 (2H, m, 2 × Ar-CH), 6.22 (1H, d, *J* = 8.3, -CONH), 4.81 (1H, ddd, *J* = 8.2, 6.9, 6.9, -CH), 3.59 (1H, s, -COCH_2_), 3.58 (1H, s, -COCH_2_), 3.17 (3H, s, -NCH_3_), 2.74 (1H, dd, *J* = 13.3, 6.9, -CH_2_), 2.54 (1H, dd, *J* = 13.3, 7.0, -CH_2_), 2.26 (3H, s, -CH_3_). 

^13^C NMR (101 MHz, CDCl_3_) δ 171.4 (-CONR_2_), 170.9 (-CONH), 142.6 (Ar-C), 136.2 (Ar-C), 135.5 (2 × Ar-C), 129.9 (2 × Ar-CH), 129.3 (2 × Ar-CH), 128.4 (Ar-C, 2 × Ar-CH), 128.2 (Ar-CH), 127.5 (2 × Ar-CH), 126.8 (Ar-CH), 121.6 (Ar-CH), 119.9 (Ar-CH), 117.9 (Ar-CH), 110.6 (Ar-CH), 104.6 (Ar-C), 51.2 (-CH), 38.9 (-CH_2_), 37.8 (-NCH_3_), 32.3 (-COCH_2_), 11.7 (-CH_3_). 

HRMS (ESI) exact mass calculated for C_27_H_27_N_3_O_2_Na [M + Na]^+^ *m*/*z* 448.1995, found *m*/*z* 448.1977. IR (thin film) 2924, 1638, 1595. 

Chiral HPLC (OD-H, 8% *i-Pr*OH in *n*-hexane, 1 mL min−1, λ 208 nm) t_Rnatural_ = 32.511 min (54.8%), t_Runnatural_ = 40.831 min (45.2%). [α${]}_{D}^{19.8{}^{\circ}C}$ +1.40 (*c* = 1.00, CHCl_3_). Preparative chiral HPLC purified (*S*)-PF74 (OD-H, 8% *i-Pr*OH in *n*-hexane, 1 mL min^−1^, λ 208 nm) t_Rnatural_ = 32.847 min (99.2%), t_Runnatural_ = 41.995 min (0.8%). [α${]}_{D}^{19.5{}^{\circ}C}$ + 520.0 (*c* = 1.00, CHCl_3_).

*Unnatural (R)-PF74* (**5b**) 

Following the general procedure described above, 70 mg (55%) of **5b** was obtained. 

^1^H NMR (400 MHz, CDCl_3_) δ 7.92 (1H, s, Ar-NH), 7.40–7.27 (5H, m, 5 × Ar-CH), 7.19–7.03 (5H, m, 5 × Ar-CH), 6.93 (2H, d, *J* = 7.0, 2 × Ar-CH), 6.66 (2H, d, *J* = 7.3, 2 × Ar-CH), 6.17 (1H, d, *J* = 8.3, -CONH), 4.78 (1H, ddd, *J* = 7.1, 7.1, 7.1, -CH), 3.58 (1H, s, -COCH_2_), 3.55 (1H, s, -COCH_2_), 3.16 (3H, s, -NCH_3_), 2.72 (1H, dd, *J* = 13.3, 6.9, -CH_2_), 2.51 (1H, dd, *J* = 13.3, 7.0, -CH_2_), 2.33 (3H, s, -CH_3_). 

^13^C NMR (101 MHz, CDCl_3_) δ 171.4 (-CONR_2_), 170.9 (-CONH), 142.6 (Ar-C), 136.2 (Ar-C), 135.5 (Ar-C), 133.5 (Ar-C), 129.9 (2 × Ar-CH), 129.3 (2 × Ar-CH), 128.4 (Ar-C), 128.4 (2 × Ar-CH), 128.2 (Ar-CH), 127.5 (2 × Ar-CH), 126.8 (Ar-CH), 121.6 (Ar-CH), 119.9 (Ar-CH), 117.9 (Ar-CH), 110.6 (Ar-CH), 104.6 (Ar-C), 51.2 (-CH), 38.9 (-CH_2_), 37.8 (-NCH_3_), 32.3 (-COCH_2_), 11.7 (-CH_3_). 

HRMS (ESI) exact mass calculated for C_27_H_27_N_3_O_2_Na [M + Na]^+^ *m*/*z* 448.1995, found *m*/*z* 448.1977. IR (thin film) 2924, 1638, 1595. 

HPLC (OD-H, 8% *i-Pr*OH in *n*-hexane, 1 mLmin^−1^, λ 208 nm) t_Rnatural_ = 32.550 min (35.0%), t_Runnatural_ = 40.678 min (65.0%). [α${]}_{D}^{20.2{}^{\circ}C}$ −5.57 (*c* = 1.00, CHCl_3_).

*Racemic PF74* (**5c**)

Following the general procedure described above, 69 mg (54%) of **5c** was obtained. 

^1^H NMR (400 MHz, CDCl_3_) δ 7.96 (1H, s, Ar-NH), 7.41–7.27 (5H, m, 5 × Ar-CH), 7.19–7.02 (5H, m, 5 × Ar-CH), 6.93 (2H, d, *J* = 6.8, 2 × Ar-CH), 6.65 (2H, d, *J* = 6.9, 2 × Ar-CH), 6.18 (1H, d, *J* = 8.3, -CONH), 4.78 (1H, ddd, *J* = 7.1, 7.1, 7.1, -CH), 3.58 (1H, s, -COCH_2_), 3.57 (1H, s, -COCH_2_), 3.16 (3H, s, -NCH_3_), 2.72 (1H, dd, *J* = 13.3, 6.9, -CH_2_), 2.51 (1H, dd, *J* = 13.3, 7.0, -CH_2_), 2.32 (3H, s, -CH_3_).

^13^C NMR (101 MHz, CDCl_3_) δ 171.4 (-CONR_2_), 170.9 (-CONH), 142.6 (Ar-C), 136.2 (Ar-C), 135.5 (Ar-C), 133.5 (Ar-C), 129.9 (2 × Ar-CH), 129.3 (2 × Ar-CH), 128.4 (Ar-C), 128.4 (2 × Ar-CH), 128.2 (Ar-CH), 127.5 (2 × Ar-CH), 126.8 (Ar-CH), 121.6 (Ar-CH), 119.9 (Ar-CH), 117.9 (Ar-CH), 110.6 (Ar-CH), 104.6 (Ar-C), 51.2 (-CH), 38.9 (-CH_2_), 37.8 (-NCH_3_), 32.3 (-COCH_2_), 11.7 (-CH_3_). 

HRMS (ESI) exact mass calculated for C_27_H_27_N_3_O_2_Na [M + Na]^+^ *m*/*z* 448.1995, found *m*/*z* 448.1977. IR (thin film) 2924, 1638, 1595. 

HPLC (OD-H, 8% *i-Pr*OH in *n*-hexane, 1 ML min^−1^, λ 208 nm) t_Rnatural_ = 32.227 min (50.4%), t_Runnatural_ = 40.120 min (49.6%). [α${]}_{D}^{19.2{}^{\circ}C}$ + 0.85 (*c* = 1.00, CHCl_3_).

### 3.3. Biological Assays—Materials and Methods

#### 3.3.1. Cells and Viruses

HEK293T suspension cells were obtained as a generous gift from Professor Massimo Palmarini (Centre for Virus Research, University of Glasgow) and were cultured in DMEM (Dulbecco’s modified Eagle’s medium) with 10% FCS (fetal calf serum) and 10 μg/mL gentamicin. The VSV-G pseudotyped single-cycle HIV-1 reporter lentiviral vector CSGW is self-inactivating, and encodes GFP expressed from an internal spleen focus-forming virus (SFFV) long terminal repeat (LTR) [22], packaged using GagPol derived from HIV-1 NL4-3 [23]. CSGW virus stocks were prepared as described previously [23], by transient transfection of HEK 293T cells, the supernatant was harvested at 48 h post transfection and clarified using a 0.45-μm-pore-size filter.

#### 3.3.2. Virus Titrations

Cells were seeded in 96-well plates (50,000 cells/well) immediately prior to infection or treatment. Briefly, suspension HEK293T cells were infected with a titrated challenge of serially diluted CSGW-containing supernatant. At 48 h post infection, cells were trypsinized and incubated at 37 °C to enable a single cell suspension and fixed (4% formaldehyde saline). The fixed cells were incubated to allow complete virus inactivation prior to analysis. Quantification of GFP positive cells (representative of GFP-reporter CSGW infection) was carried out using flow cytometry with 10,000 events acquired per well (Guava EasyCyte cytometer). From the titration performed with CSGW, the concentration of virus-containing supernatant required to produce 15–20% infection of cells was calculated. This dilution was then used in in fixed CSGW dose assays.

#### 3.3.3. Compound Fixed Dose (Virus Titration) Assay

For fixed compound dose assays, a CSGW titration as described above was performed with one exception. The final addition of 50 µL fresh media described above was substituted with the addition of 50 µL of a 4% DMSO solution of compound (at 4 × desired final concentration) in media. The plates were then incubated for 48 h before being fixed (100 µL 4% formaldehyde saline) and analysed by flow cytometry.

#### 3.3.4. Compound Dose Response (Fixed Virus Dose) Assay

For compound dose response assays, each compound was titrated across a 96-well plate to give a 50 µL solution with a maximum final concentration of 100 µM to a minimum final concentration of 95 pm (×4 dilution series). In addition to the compound, each well contained 100 µL of a suspension of HEK293T cells in fresh media (50,000 cells/well) and 50 µL of diluted CSGW (at the titre previously calculated to produce ~15–20% infection) in fresh media with a final concentration of 1% DMSO per well. The plates were then incubated for 48 hours before being fixed (100 µL 4% formaldehyde saline) and analysed by flow cytometry.

### 3.4. Molecular Docking Methods

All structure preparation and docking was performed using the Schrödinger suite (build 2019-1) The enantiomers of PF74 were drawn with ChemDraw and converted to a 3D structure and energy minimized using the OPLS3e forcefield in LigPrep (Schrödinger Release 2019-1: LigPrep, Schrödinger, LLC, New York, NY, USA 2019). The hexameric quaternary structure of capsid PDB: 2XDE [5] was generated in Pymol (The PyMOL Molecular Graphics System, Version 2.0 Schrödinger, LLC, Schrödinger, Inc., New York, NY, USA) and used for docking. Protein Prep Wizard (Schrödinger Release 2019-1: Protein Preparation Wizard; Epik, Schrödinger, LLC, New York, NY, USA 2019) was used to prep PDB structures for Prime (Schrödinger Release 2019-1: Prime, Schrödinger, LLC, New York, NY, USA 2019) water molecules were kept and minimized. Molecular docking was performed using Glide and the OPLS3e force field (Schrödinger Release 2019-1: Glide, Schrödinger, LLC, New York, NY, USA 2021). Molecular Mechanics/Generalized Born Surface Area (MMGBSA) protein–ligand interaction energies were calculated for the top poses of both enantiomers in a rigid receptor with the VSGB solvent model and the OPLS3e forcefield (Schrödinger Release 2019-1: Maestro, Schrödinger, LLC, New York, NY, USA 2019).

## 4. Conclusions

To summarise, our improved syntheses of (*S*)-, (*R*)- and racemic PF74 were based on a previously published procedure [13]. Chiral HPLC analyses of the synthesised materials were conducted, which established a significant degree of epimerisation was present in each batch. Preparative chiral HPLC was utilised to generate highly enantiomerically enriched (≥98%) samples of (*S*)- and (*R*)-PF74. Evaluation of the biological activities was conducted in HEK293T cells to assess the ability of each enantiomer to inhibit the infectivity of the HIV viral vector CSGW-VSV-G. In broad agreement with recently published work [14], this testing determined (*S*)-PF74 to be significantly more active (IC_50_ = 1.5 µM) than (*R*)-PF74 (IC_50_ = 19 µM). In an effort to rationalise this observation, docking studies were conducted which showed the two enantiomers adopted different conformations in the PF74 binding site. The more active (*S*)-PF74 had additional favourable interactions compared to (*R*)-PF74, which was reflected in the calculated binding energies of each conformation, the more active (*S*)-PF74 (ΔG of −73.8 kcal/mol) being significantly lower in energy than (*R*)-PF74 (ΔG of −55.8 kcal/mol).

## Data Availability

Not applicable.

## Supplementary Materials

The following are available online: NMR spectra.

## Sample Availability

Samples of compounds are not available from the authors.
