# Supplementary material for: Synthesis, Enantiomeric Resolution and Biological Evaluation of HIV Capsid Inhibition Activity for Racemic, (S)- and (R)-PF74
Source: Molecules. 2021 Jun 26;26(13):3919. doi: 10.3390/molecules26133919 (PMC8272108; doi:10.3390/molecules26133919)

Supplementary Material

Synthesis, Enantiomeric Resolution and Biological Evaluation of HIV Capsid Inhibition Activity for Racemic, (*S*)- and (*R*)-PF74.

^{1}H NMR Spectra of methyl ester **3a**

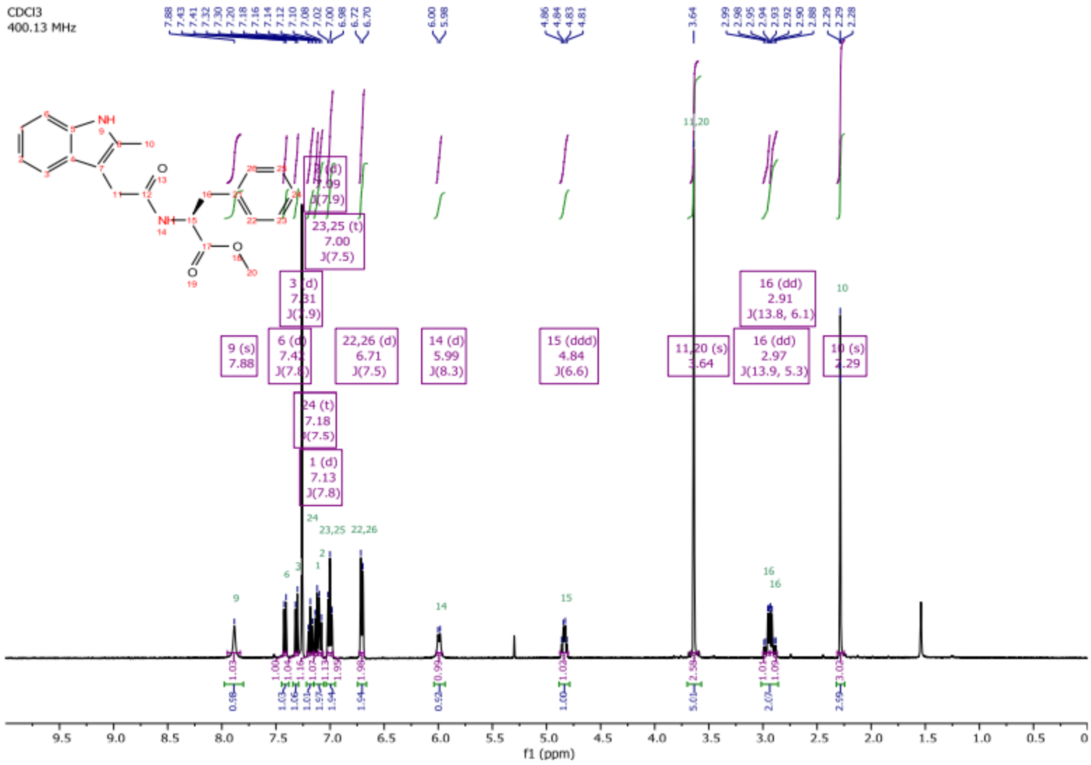

^{13}C NMR Spectra of methyl ester **3a**

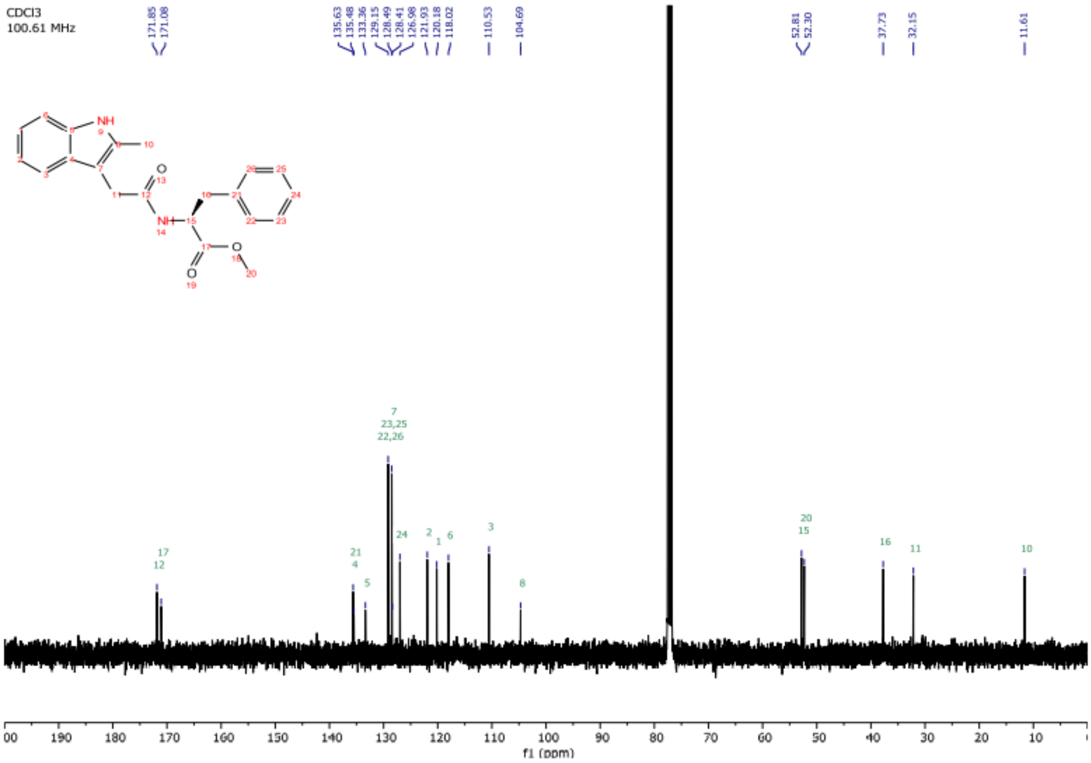

^{1}H NMR Spectra of methyl ester **3b**

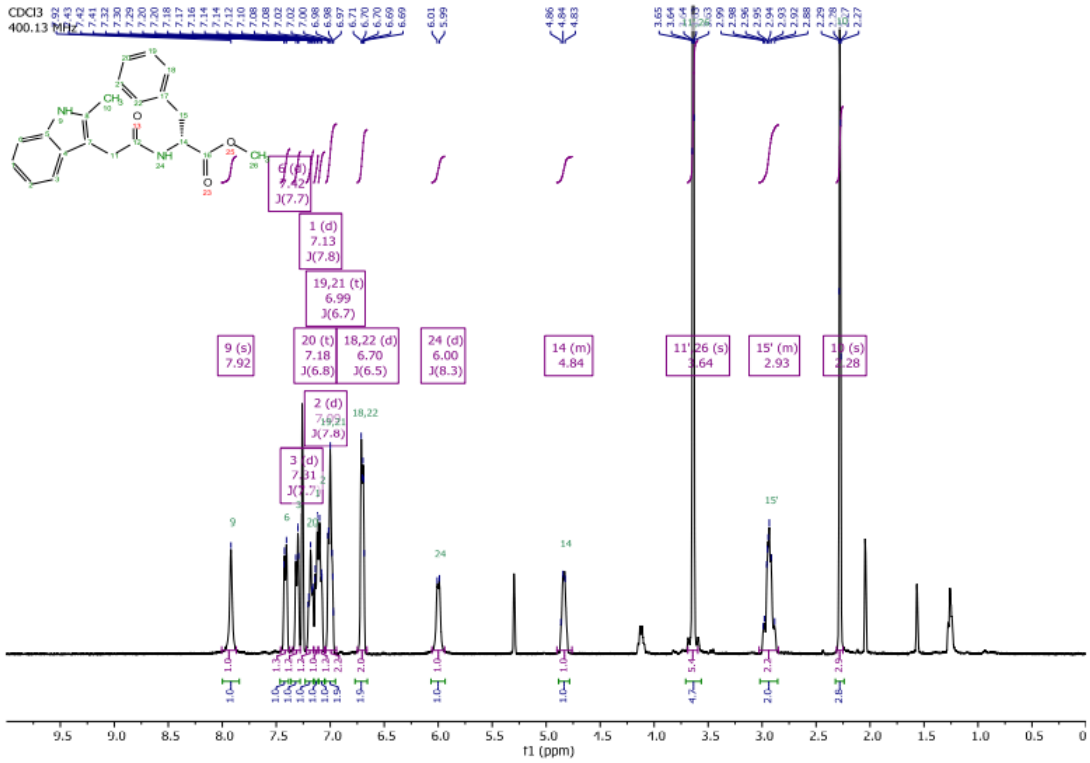

^{13}C NMR Spectra of methyl ester **3b**

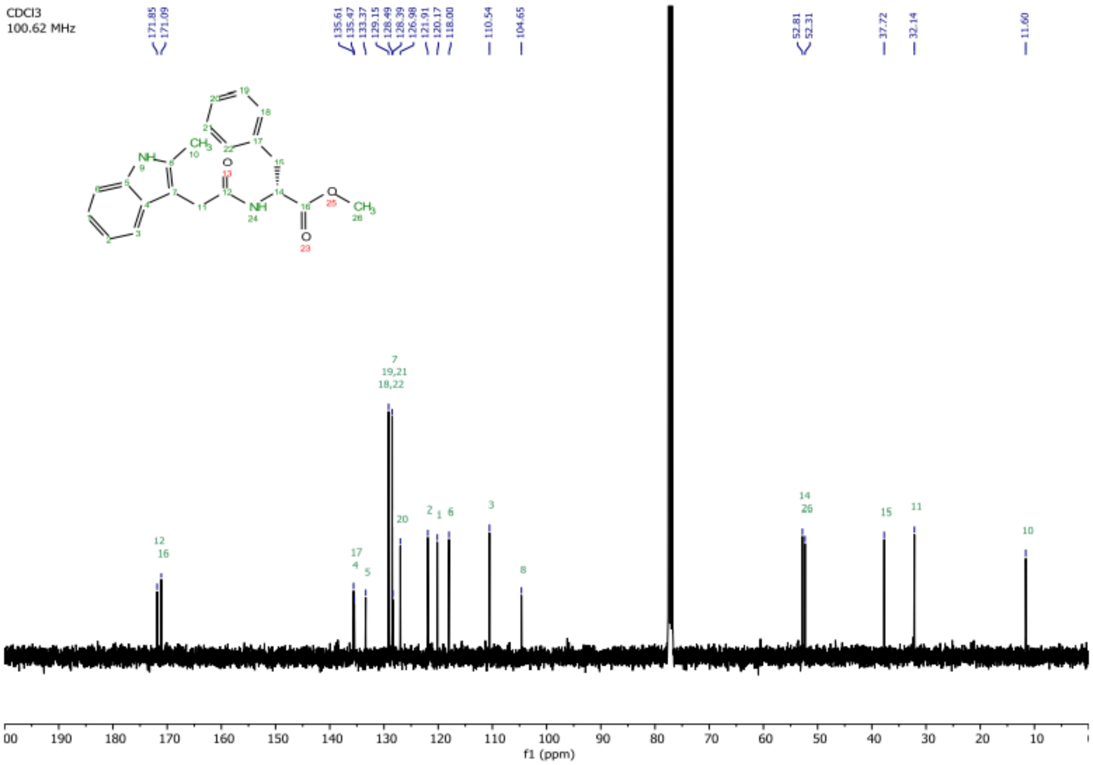

HSQC NMR Spectra of methyl ester **3b**

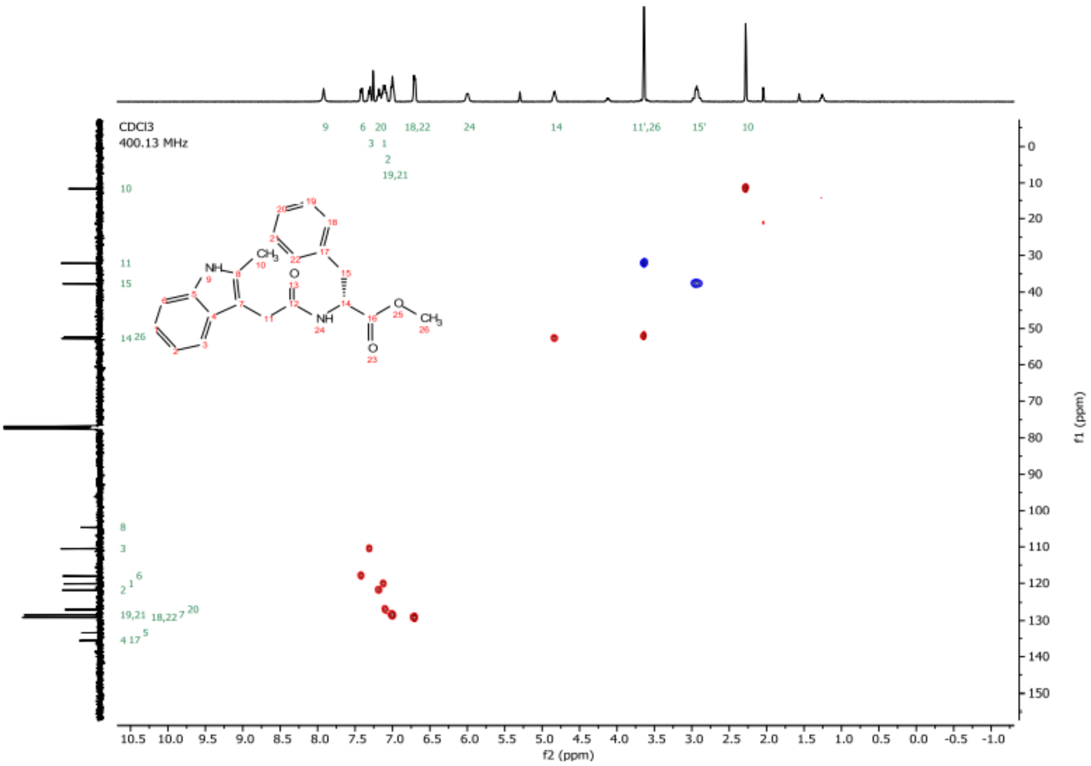

^{1}H NMR Spectra of methyl ester **3c**

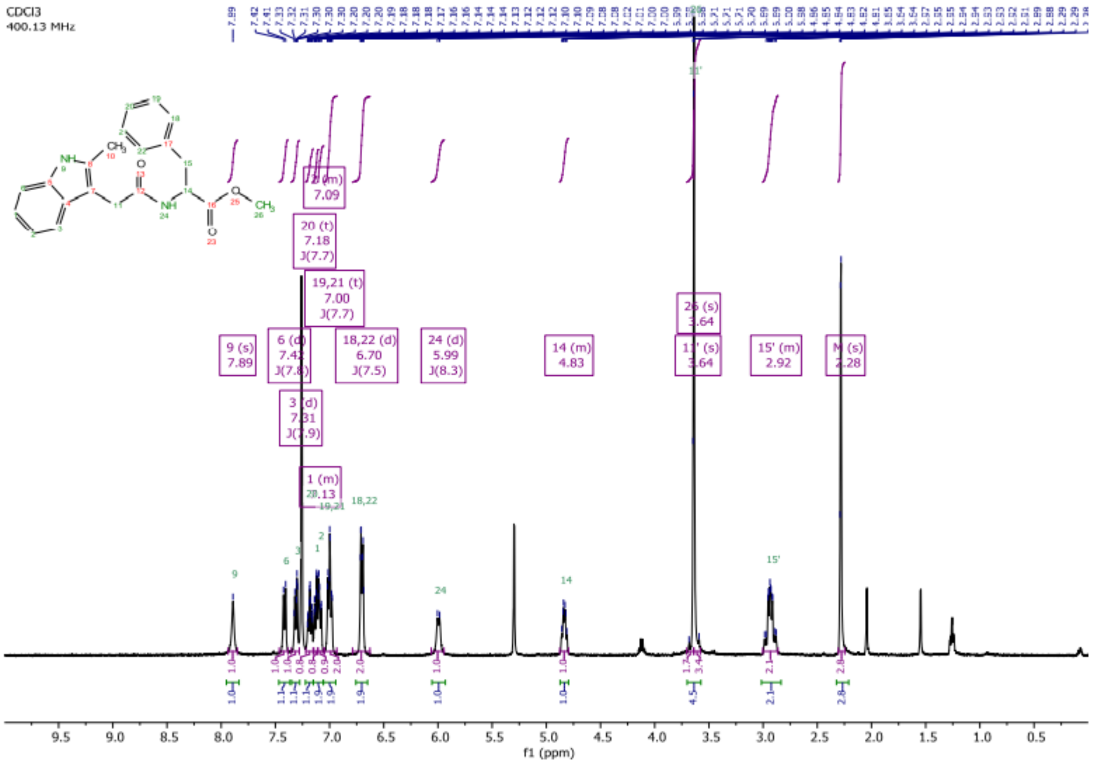

^{1}H NMR Spectra of acid **4a**

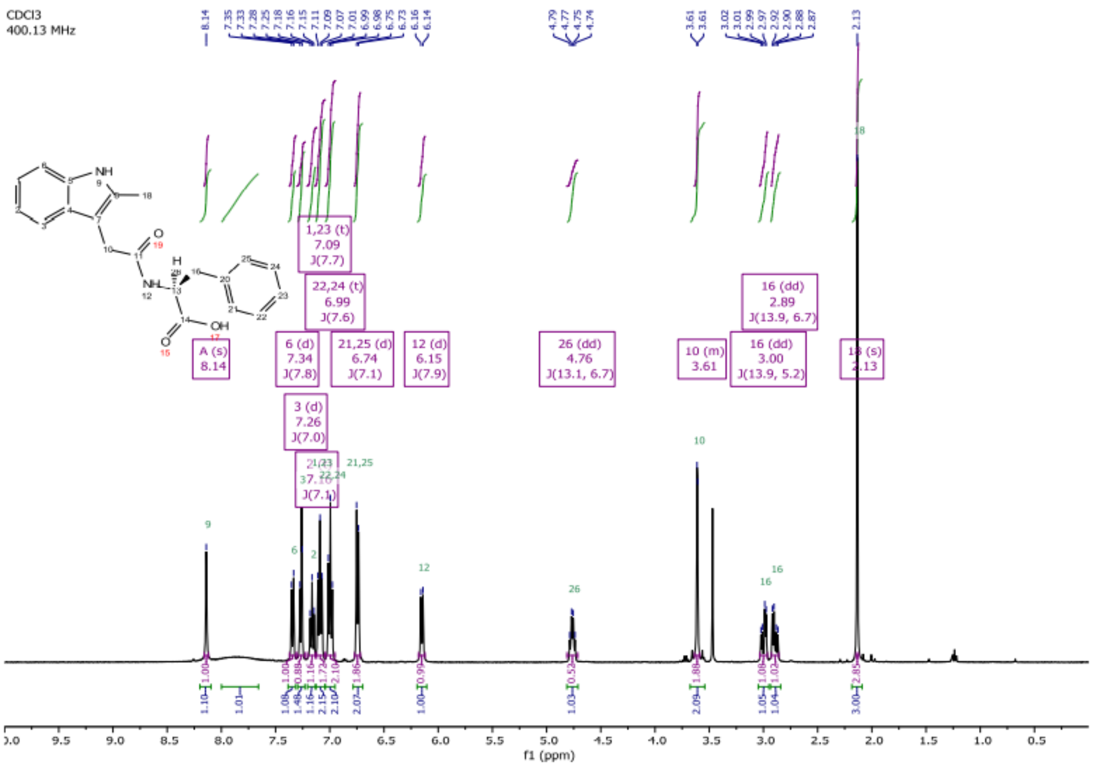

^{13}C NMR Spectra of acid **4a**

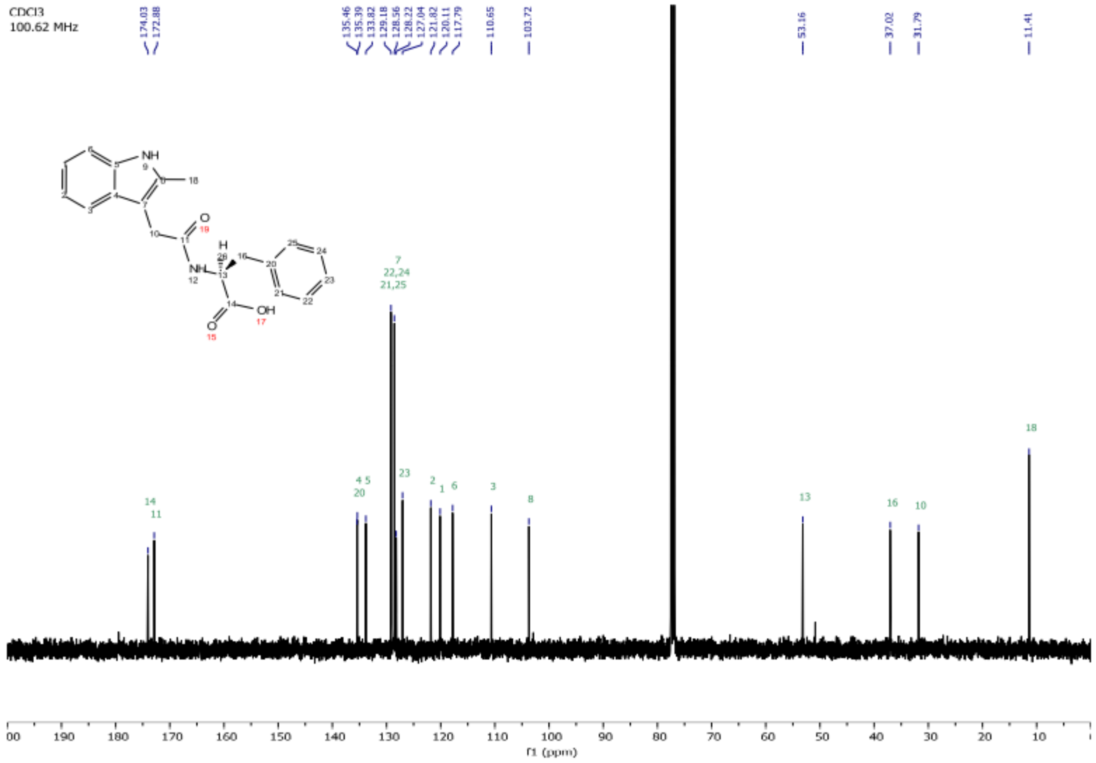

COSY NMR Spectra of acid **4a**

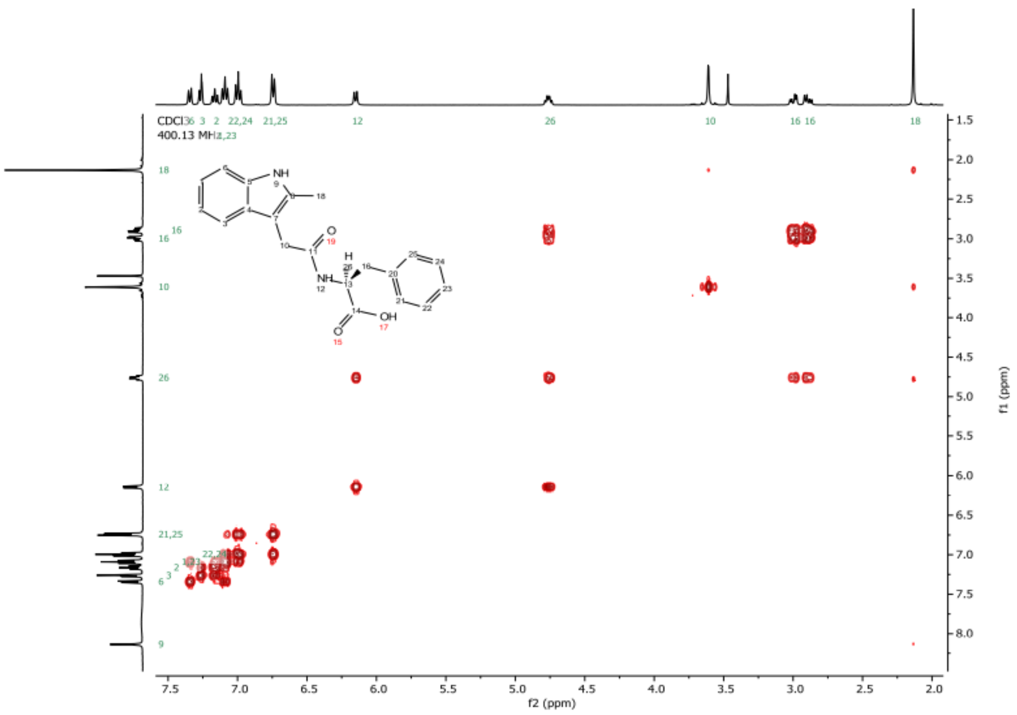

DEPT-135 NMR Spectra of acid **4a**

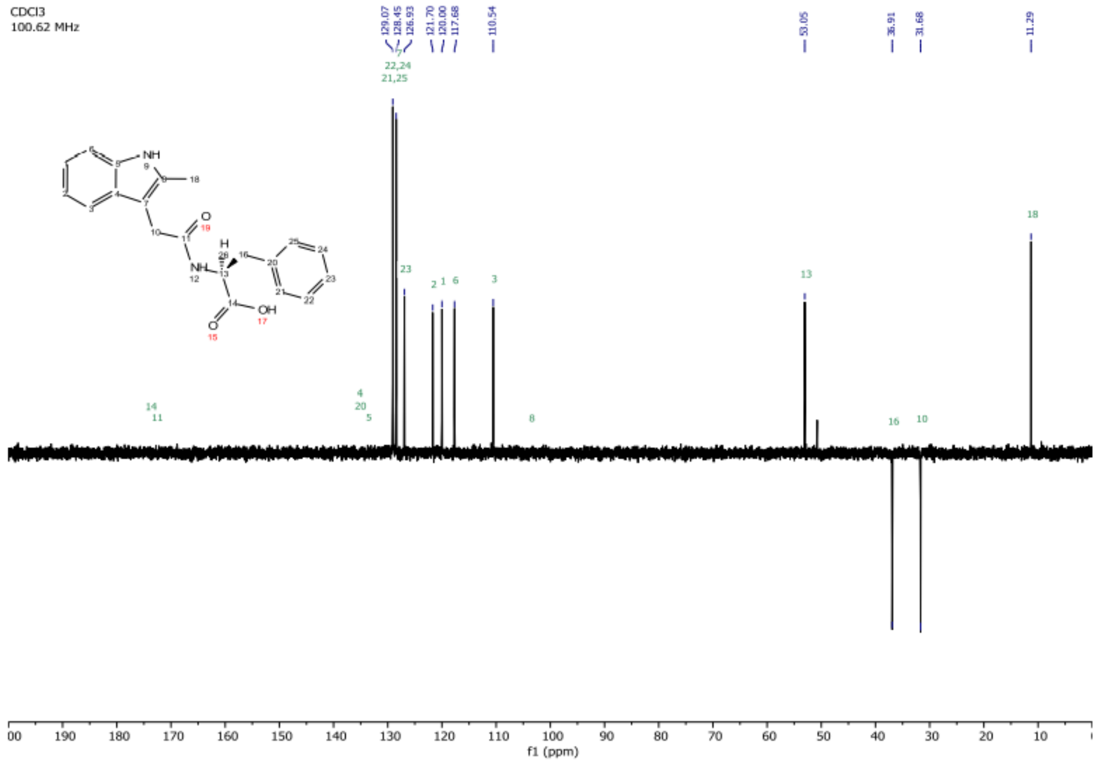

HSQC NMR Spectra of acid **4a**

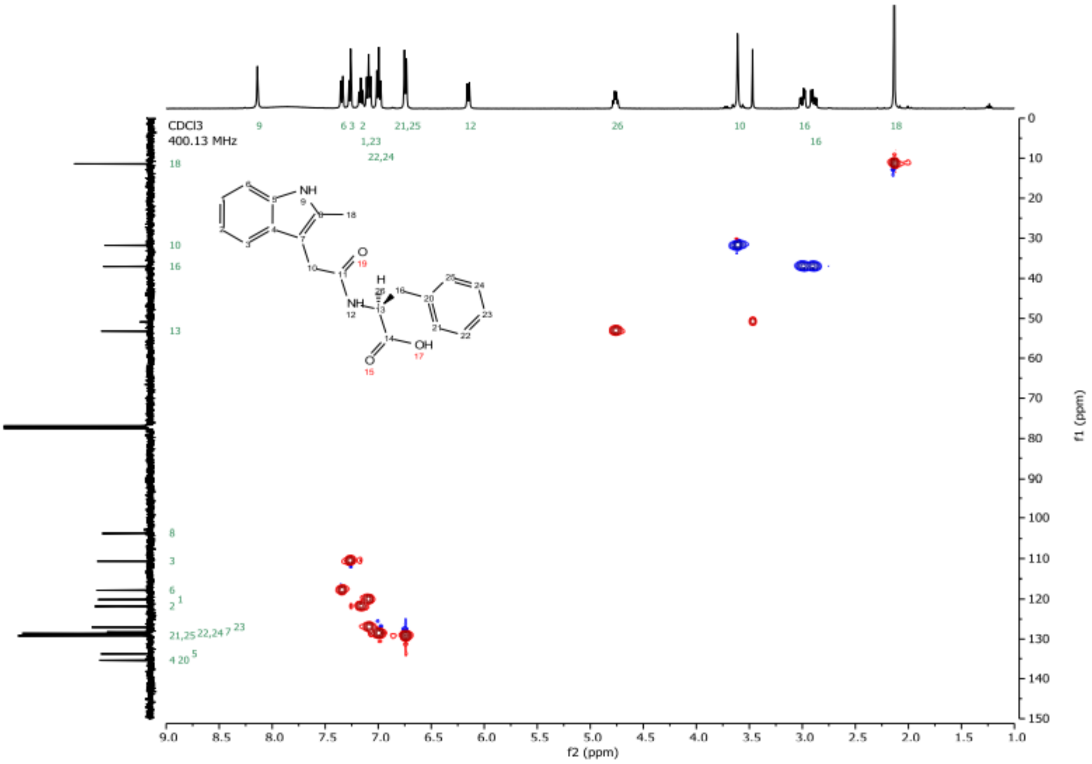

HMBC NMR Spectra of acid **4a**

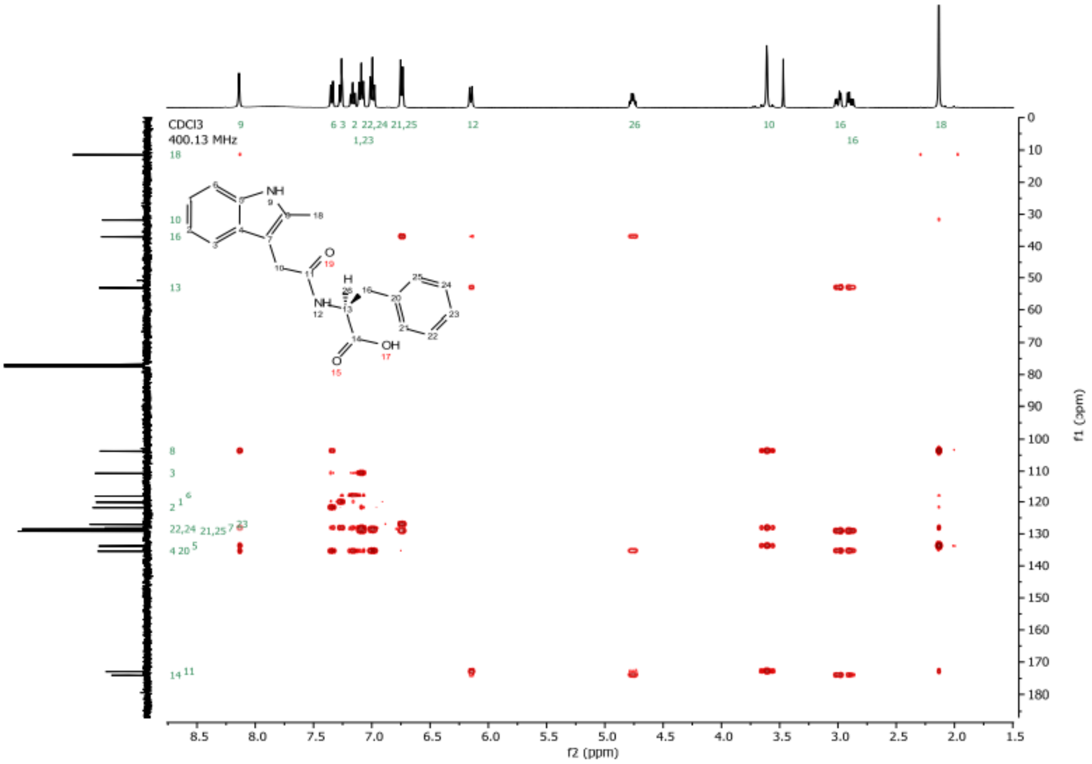

^{1}H NMR Spectra of acid **4b**

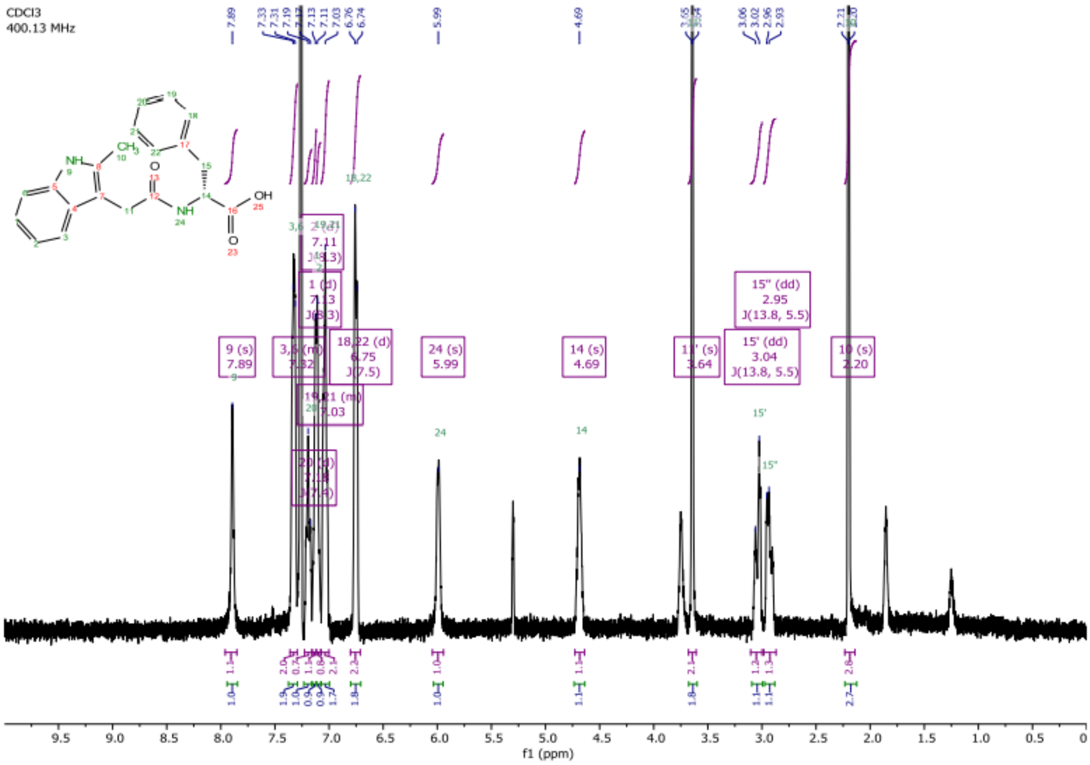

^{1}H NMR Spectra of acid **4c**

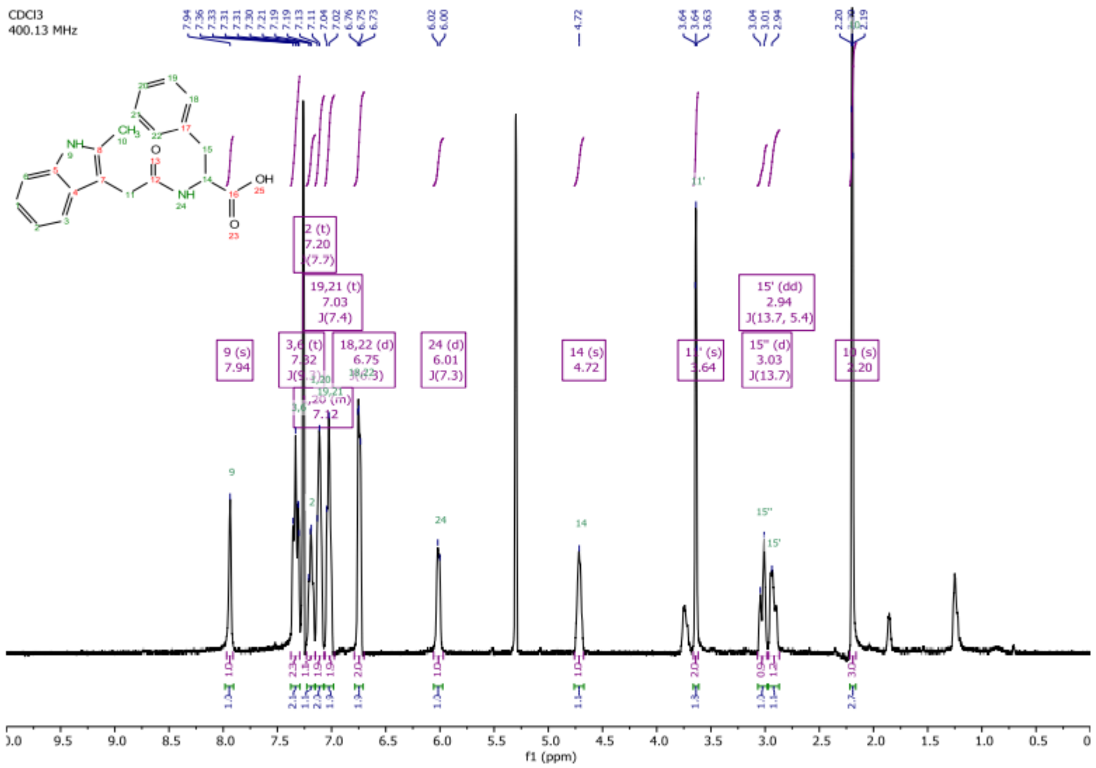

^{1}H NMR Spectra of natural PF74 **5a**

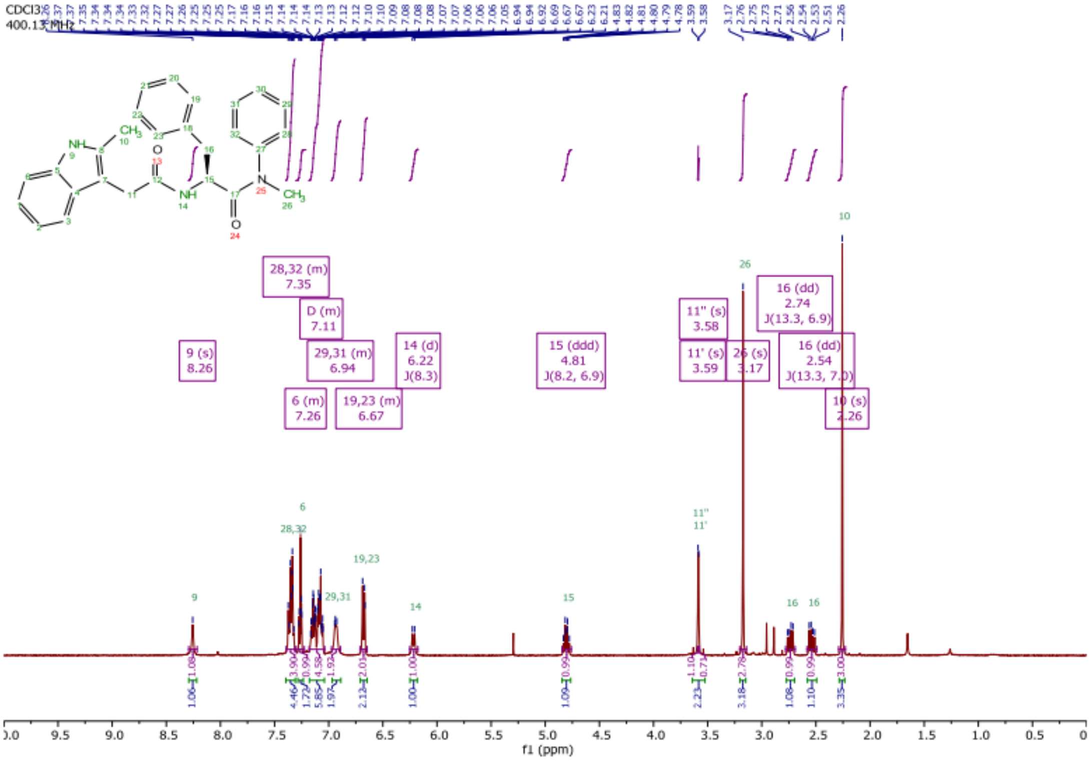

^{13}C NMR Spectra of natural PF74 **5a**

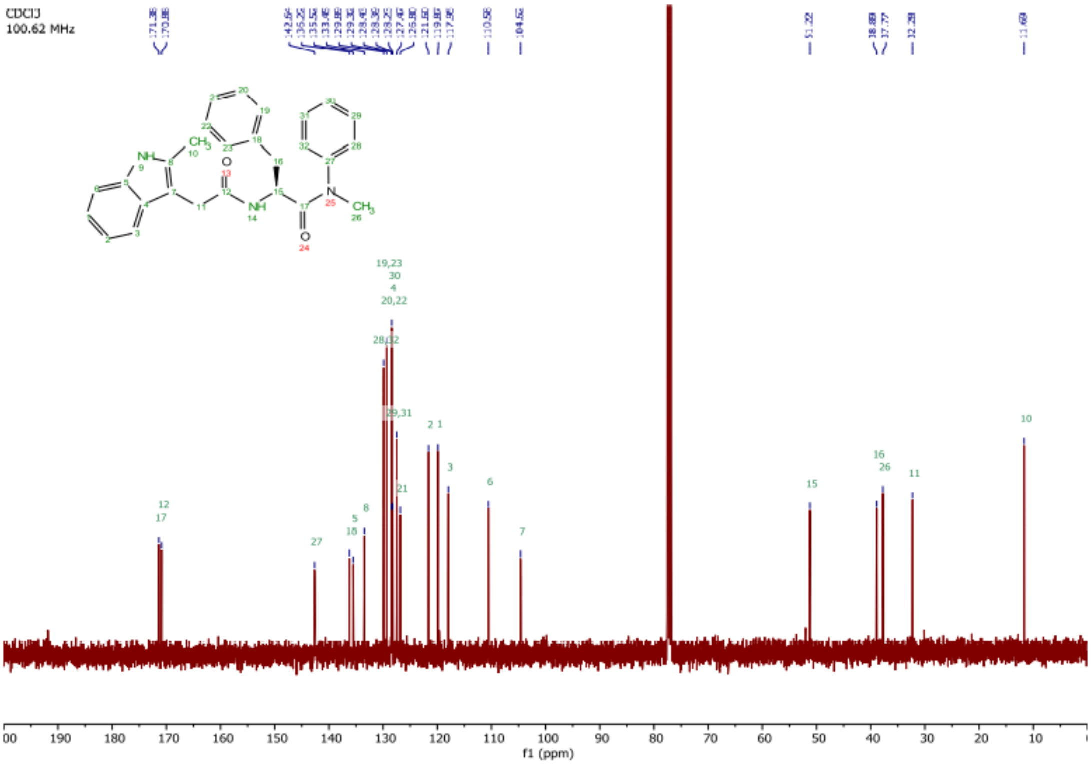

COSY Spectra of natural PF74 **5a**

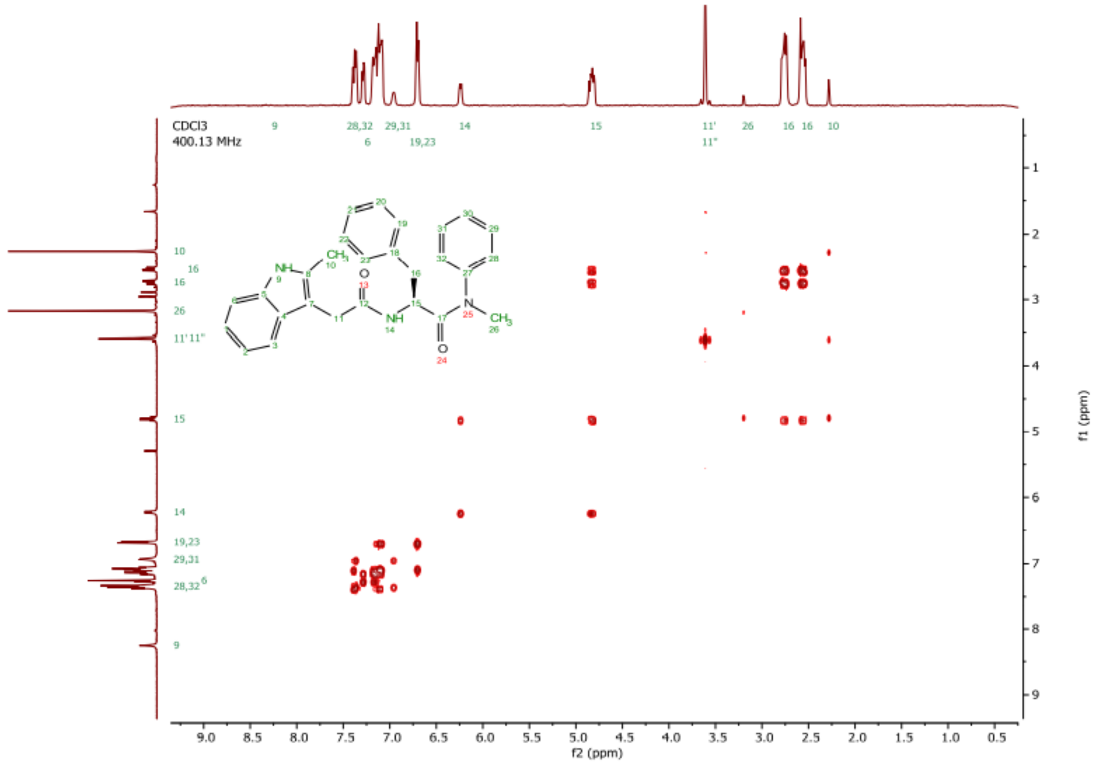

HSQC Spectra of natural PF74 **5a**

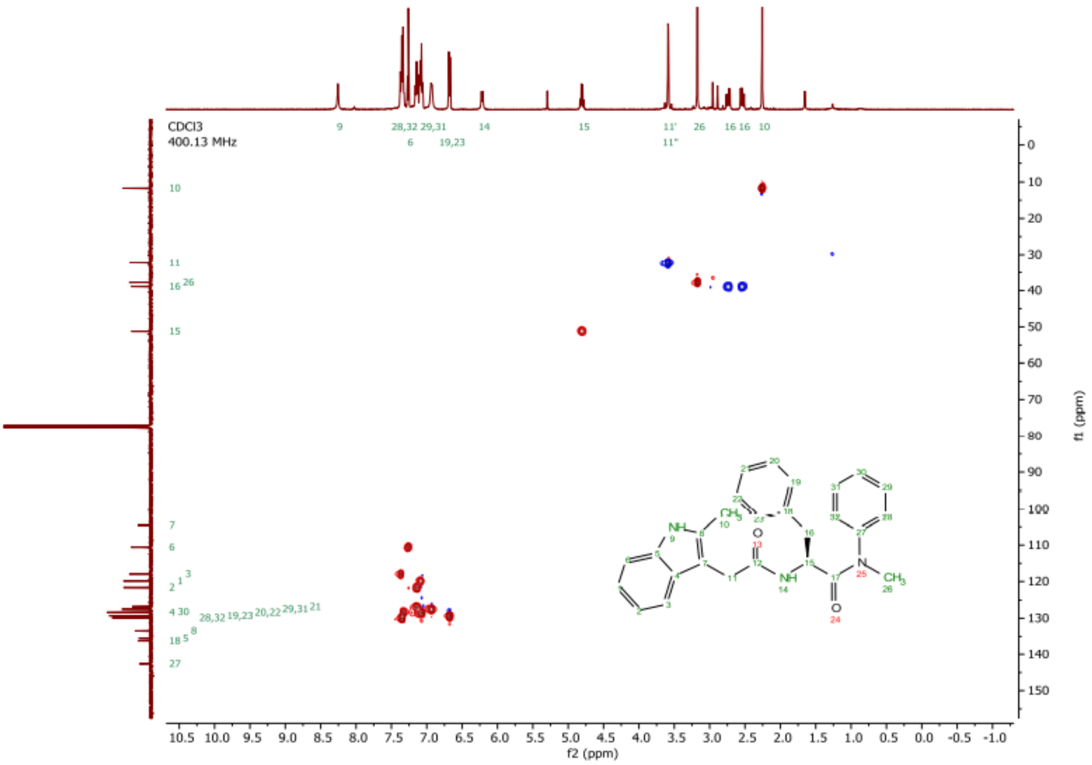

HMBC Spectra of natural PF74 **5a**

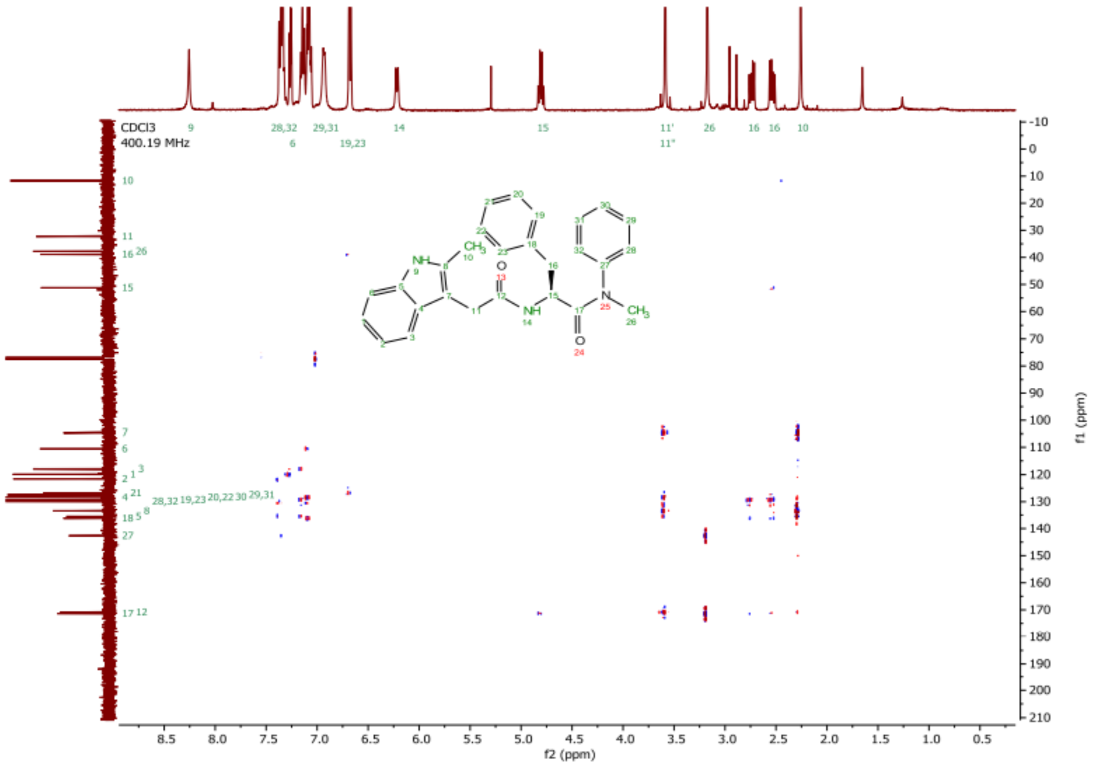

^{1}H NMR Spectra of unnatural PF74 **5b**

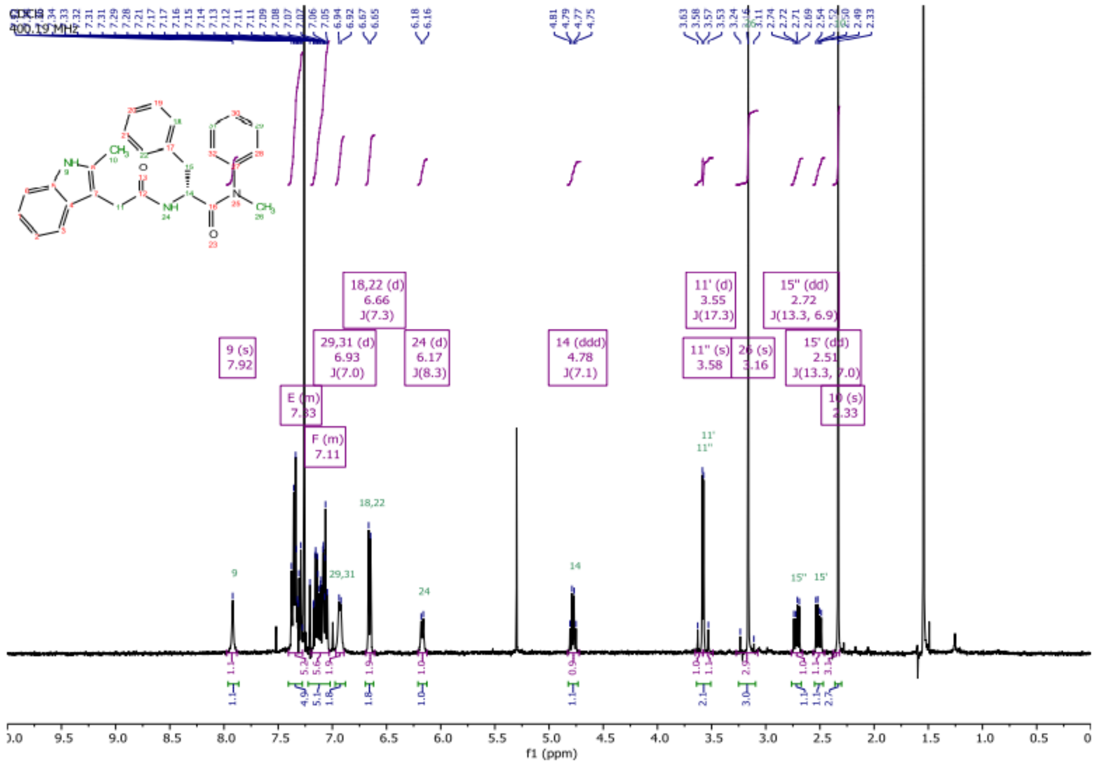

^{1}H NMR Spectra of racemic PF74 **5c**

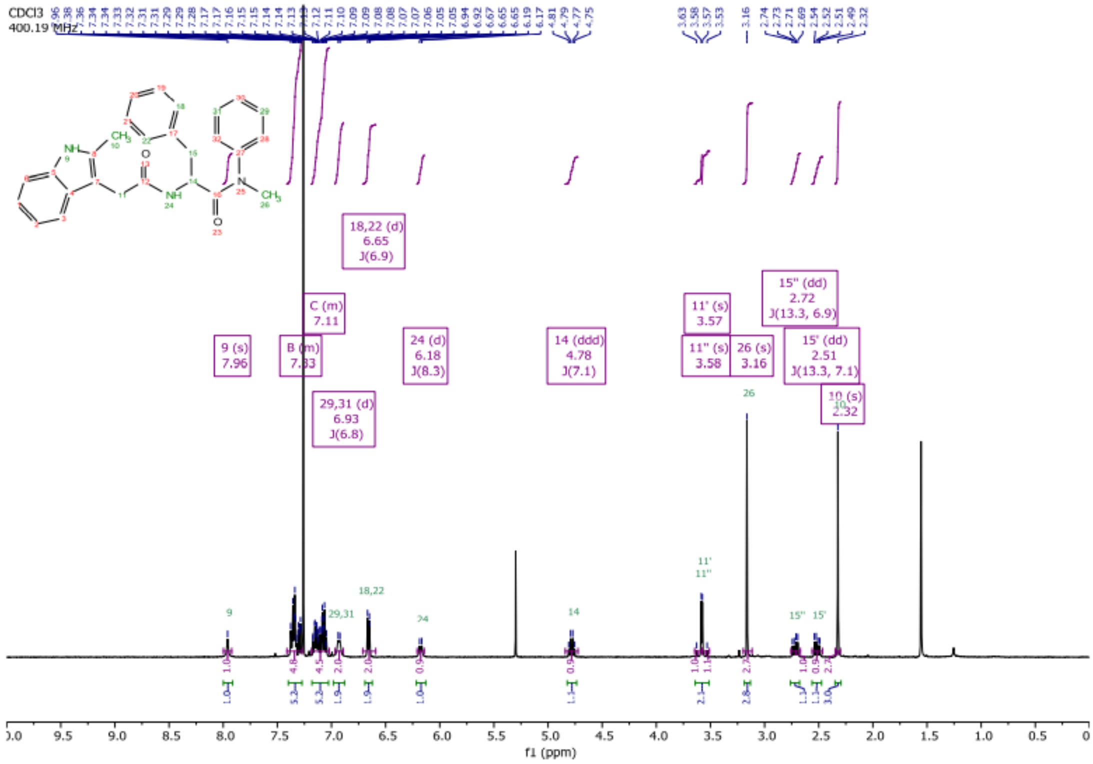

Supplement: Supplementary file 1 [file molecules-26-03919-s001.zip › molecules-1262644-supplementary.pdf]
